# Father Involvement and Children’s Social Behavior: A Three-Level Meta-Analysis

**DOI:** 10.3390/bs16081426

**Published:** 2026-08-19

**Authors:** Yiyang Li, Jun Hao

**Affiliations:** College of Teacher Education, Ningbo University, Ningbo 315211, China; 2311030212@nbu.edu.cn

**Keywords:** father involvement, positive social behavior, negative social behavior, meta-analysis

## Abstract

The relationship between father involvement and child development has received increasing attention. This study employed a three-level meta-analytic model with the aims of clarifying the relationship between father involvement and children’s social behaviors (positive/negative) and analyzing the factors influencing the differences in this relationship. Through literature retrieval and screening, 37 studies were included in this research, and a total of 249 effect sizes were extracted. The results of the meta-analysis show that there is a moderate positive correlation (*r* = 0.203) between father involvement and children’s positive social behavior, and a low negative correlation (*r* = −0.087) between father involvement and children’s negative social behavior. This relationship was significantly moderated by children’s age stage, family socioeconomic status, type of father involvement, reporter of father involvement, cultural background and study quality, while children’s gender exerted no moderating effect. These findings highlight the importance of the father’s role in the development of children’s social behaviors. Future research could further focus on the quality of father involvement in different cultural backgrounds and the impact of mother involvement on children’s development.

## 1. Introduction

In recent decades, there has been a growing global recognition of fathers’ unique and multifaceted contributions to children’s development. Socially, evolving gender attitudes have reshaped cultural expectations of masculinity and family roles. As the conventional “breadwinner-only” paternal norm gradually fades, society has become more receptive to fathers taking an active and direct part in childcare (Lamb, 2010). Concurrently, expanding policy support for caregiving across many national contexts, such as the rollout of paid paternity leave quotas, father-targeted family benefits, and work-family reconciliation policies, has further legitimized and incentivized paternal engagement in direct care work (Streckenbach et al., 2022).

Father involvement (FI) refers to fathers’ multifaceted engagement with their children, encompassing a broad range of behaviors and interactions related to caregiving, developmental activities, and interpersonal exchanges, regardless of their positive or negative valence (Lamb et al., 1985; Pleck, 2010). Extensive research has demonstrated that FI is associated with children’s physical health, mental health, cognitive development, academic achievement, social adjustment and other domains (Cano et al., 2019; Flouri & Buchanan, 2003; Jeynes, 2015; Paquette et al., 2023; W. Wu et al., 2023).

Numerous studies have demonstrated a significant association between father involvement and children’s social behavior. On the one hand, fathers’ active participation has been shown to foster children’s prosocial behaviors such as helping and cooperation, which benefit others and society. Father involvement has been linked to higher levels of children’s social competence (Torres et al., 2014), has promoted the development of prosocial tendencies (van der Storm et al., 2022) and has mitigated social withdrawal (Amaral et al., 2019). On the other hand, father involvement has also exhibited negative impacts on social behavior development. A recent meta-analysis has revealed that fathers’ negative involvement is associated with increased problematic behaviors in children (J. Zhang et al., 2021), while the absence of father involvement or adoption of negative parenting styles may exacerbate children’s negative social behaviors such as aggression and withdrawal (Jaffee et al., 2003).

While a large body of quantitative research has examined the link between FI and children’s social behavior over recent decades, findings have not been uniformly consistent. Cumulative evidence generally points to a positive association between FI and children’s positive social behavior (Khawaja et al., 2024; Ramchandani et al., 2010; W. Wu et al., 2023), as well as a negative correlation between FI and children’s negative social behavior (Levant et al., 2014; X. Wang et al., 2019; Yoon et al., 2025). However, notable inconsistencies have emerged across individual studies: for example, fathers’ use of physical punishment and inconsistent discipline has been linked to lower levels of children’s prosocial behavior (Gryczkowski et al., 2018), while other studies have found fathers’ emotional support and behavioral control to correlate with increased aggressive tendencies in children (Bagán et al., 2019). These mixed results underscore the need to clarify the nature of this relationship further.

Beyond these conflicting findings, existing research on FI and children’s social behavior suffers from several key limitations. First, the literature lacks systematic integration of different forms of father involvement, often treating the construct as unidimensional rather than multifaceted. Second, prior work has often failed to account for critical contextual factors, including children’s age and gender, family socioeconomic status, reporter bias, and cross-cultural differences between Eastern and Western contexts. Moreover, previous meta-analyses on this topic have been largely restricted to unidimensional positive social behavior outcomes (van der Storm et al., 2022) and have not comprehensively synthesized evidence for both positive and negative social behaviors.

Therefore, the inconsistent findings and methodological gaps in prior research justify the need for the present comprehensive meta-analysis to integrate conflicting findings and provide more reliable evidence. This study aims to employ meta-analytic methods to systematically examine the association between FI and children’s social behavior (both positive and negative), and to investigate the contextual factors that may moderate this relationship.

### 1.1. Potential Moderators

#### 1.1.1. Children’s Gender

As an individual characteristic of children, gender may be a key moderating factor. Previous studies have found that girls tend to display more prosocial behaviors than boys, while boys often exhibit higher levels of aggressive behavior (Bagán et al., 2019). Fathers are generally more engaged with boys than with girls (Yeung et al., 2001) and tend to have a greater impact on boys’ social development (Aldous & Mulligan, 2002; Chang et al., 2003). This suggests that gender differences may not only influence children’s social behavior directly but also shape developmental outcomes by affecting the type and degree of father involvement. However, some studies have argued that fathers’ involvement does not differ significantly by children’s gender (Carlson, 2006; X. C. Wu et al., 2014), and that gender does not moderate the link between fathers’ parenting behaviors and children’s physical aggression (Kawabata & Crick, 2016). These conflicting findings indicate that prior research has produced inconsistent results. Therefore, we incorporate children’s gender as a moderating variable.

#### 1.1.2. Children’s Age Stage

Previous studies have found that children’s age may moderate the relationship between father involvement and children’s social behaviors. For example, fathers’ participation in children’s daily life care decreases as children age, while their involvement in rule-setting and behavioral supervision of school-age children increases (X. C. Wu et al., 2014; Xu, 2010). This variation can be attributed to the fact that the specific content and extent of father involvement differ across various age stages. Given that prior research has primarily focused on children aged 3 to 12 years, a broad age spectrum, this study categorizes children into preschool (3–6 years) and school-aged (7–12 years) groups and examines these as moderating variables.

#### 1.1.3. Family Socioeconomic Status

Numerous studies have demonstrated a significant association between family socioeconomic status (SES) and children’s developmental outcomes, though findings remain inconsistent. Early research has shown that fathers in high-SES families are more engaged and provide their children with abundant material resources and educational support (Carlson & Magnuson, 2011), leading to better developmental outcomes. In contrast, fathers in low-SES families have been found to be less involved and more likely to exhibit negative parenting behaviors (McLoyd, 1998). With children from low-SES backgrounds experiencing higher rates of psychological issues (Davis et al., 2010). However, recent studies have suggested that SES may not moderate the link between father involvement and problem behaviors in preschoolers (J. Zhang et al., 2021). Notably, the positive impact of father involvement on academic performance has been found to be significantly stronger for children from low-SES families than for those from middle- and high-SES families (Miller et al., 2020). These divergent findings indicate that the relationship among SES, father involvement and children’s development is unlikely to be linear. Therefore, we incorporate family SES as a moderating variable.

#### 1.1.4. Type of Father Involvement

Empirical research has shown that different types of father involvement are associated with distinct dimensions of children’s social behavior development. For example, fathers’ positive involvement and warmth have been linked to higher social competence and prosocial behaviors, whereas neglect and corporal punishment are associated with increased aggression and social withdrawal (Bagán et al., 2019; Gryczkowski et al., 2018; Jaffee et al., 2003). This disparity may stem from the conceptual complexity of father involvement and the diversity of involvement patterns. Existing research has highlighted multiple dimensions of father involvement: Lamb (1975) emphasized the significance of direct caregiving as a form of general involvement, while Hawkins et al. (2002) expanded this framework by examining emotional support and rule guidance, introducing constructs such as supportive involvement and non-coercive control. More recently, studies have acknowledged the potential negative dimensions of father involvement, including neglect, rejection, and inconsistent parenting (Amato & Rivera, 1999). Drawing on established conceptual frameworks and prior meta-analyses of father involvement (J. Zhang et al., 2021), this study categorizes father involvement into four distinct types: general involvement (participation in daily life, learning, play, and financial support), supportive involvement (active responsiveness, praise, respect, and emotional warmth), non-coercive control (disciplinary rule-setting and supervision), and negative involvement (punishment, neglect, rejection, hostility, and inconsistency). This typology aims to provide a clear framework for dissecting the multifaceted nature of father involvement. Given that these four types differ in interaction quality, involvement dynamics, and influence pathways, their effects on children’s social behaviors are likely to vary significantly. By incorporating father involvement types as moderating variables, this study seeks to not only reflect the multidimensional complexity of father involvement but also uncover the differential impact mechanisms of distinct involvement patterns on children’s social behaviors.

#### 1.1.5. Reporter of Father Involvement

Reporters may serve as moderating variables influencing the relationship under study. Existing research has shown that fathers’ self-reported involvement levels are significantly higher than those of mothers (Kitterød & Lyngstad, 2014). Moreover, measurements by a single reporter tend to yield less accurate results compared to multi-reporter assessments (De Los Reyes et al., 2015). This discrepancy may stem from differences in information acquisition perspectives, evaluation biases and sensitivity to involvement types across reporters. These variations suggest that when different reporters assess the same type of father involvement, the strength and direction of its association with children’s social behavior may differ. Therefore, we incorporate the reporter of father involvement as a moderator variable.

#### 1.1.6. Cultural Background

Cultural background differences may influence the relationship between father involvement and children’s social behaviors. For instance, research has indicated that father involvement levels in Indian families are significantly higher than those in British white families (Flouri, 2005). Moreover, parenting styles in Eastern cultures tend to be more strict (J. Yang & Zhao, 2020). This disparity can be attributed to divergent cultural values between the East and West, which shape distinct parenting practices. Consequently, these cultural variations may give rise to cross-cultural differences in how father involvement influences children’s social behavior mechanisms. Therefore, we examine cultural background as a moderating variable.

#### 1.1.7. Study Quality

Study quality differences may influence the relationship between father involvement and children’s social behaviors. For instance, meta-analytic research has indicated that lower-quality primary studies often report larger effect sizes than higher-quality studies, potentially due to methodological limitations such as measurement bias, small sample sizes, or selective reporting (Lipsey & Wilson, 2001). Consequently, these methodological variations may give rise to differences in how father involvement correlates with children’s social behavior across studies. Therefore, we examine study quality as a moderating variable.

### 1.2. The Present Study

This study conducts a meta-analysis of existing empirical research to systematically explore the association between father involvement and children’s social behaviors (positive/negative) and the potential moderating variables. This study aims to test the following research hypotheses:

1. Father involvement is significantly positively correlated with children’s positive social behavior and significantly negatively correlated with children’s negative social behavior;
2. Children’s gender, age stage, family socioeconomic status, the type of father involvement, the reporter of father involvement, cultural background and study quality significantly moderate the relationship between father involvement and children’s social behaviors (positive/negative).

## 2. Materials and Methods

### 2.1. Literature Search

This study utilized several databases for data retrieval: Web of Science, Scopus, PubMed, Wiley Online Library, PsycINFO via EBSCOhost and China National Knowledge Infrastructure (CNKI). Since research on father involvement began in the 1970s, the retrieval period was set from January 1970 to May 2025. The search terms included: 1. father involvement, father engagement, paternal involvement; 2. social behavior, prosocial behavior, aggressive behavior, aggression, withdrawal, shyness; 3. children and child. The search formula was constructed using Boolean logical operators (AND/OR) and supplemented by reference literature tracing. Finally, the search formula for the databases was: (“paternal involvement” OR “father involvement” OR “father participation” OR “father engagement”) AND (“social behavior” OR “prosocial behavior” OR “aggressive behavior” OR “aggression” OR “withdrawal” OR “shy”) AND (children*), English for international databases, Chinese for CNKI (The full search strategies for all databases are available in the Supplementary Materials).

### 2.2. Inclusion Criteria

The following criteria were applied to select studies:

1. It must be a published research report, excluding dissertations and other document types (conference proceedings, book chapters, reports, preprints) to ensure the quality of peer review.
2. It must be an empirical study, excluding theoretical and literature review articles.
3. It should report in detail at least the r value between one dimension of father involvement and one type of children’s social behaviors, or the F value, β value, t value, or χ^2^ value that can be converted into an r value.
4. The average age of children in the study should be between 3 and 12 years old.
5. Only children from the general population (children without confirmed clinical diagnoses of disabilities, neurodevelopmental disorders, or enrollment in formal special education/intervention programs) are included. No children are excluded based on ethnic or cultural background.

**Figure 1:**
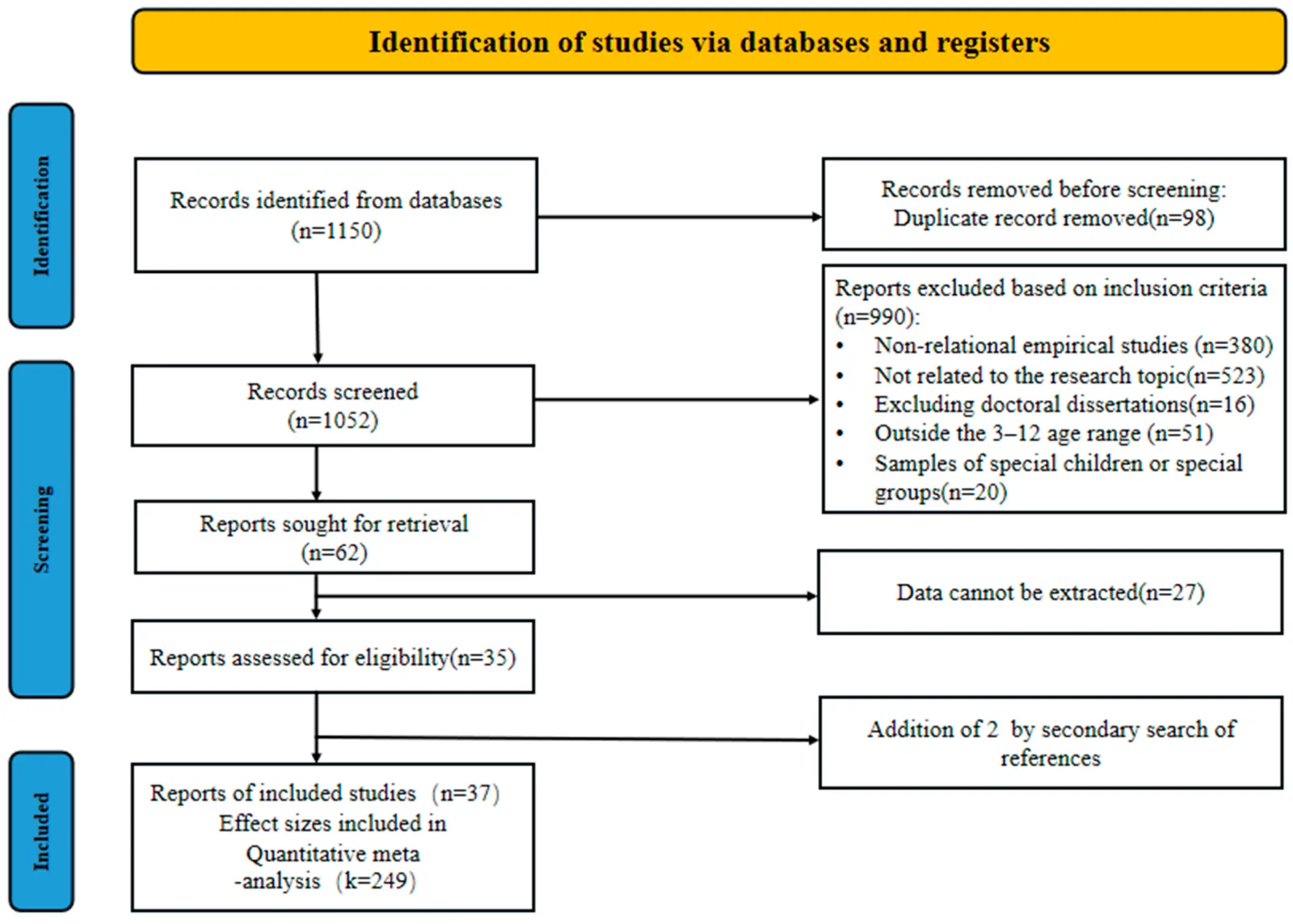
PRISMA diagram.

The PRISMA diagram of our literature search (Page et al., 2021) is presented in Figure 1.

### 2.3. Literature Coding and Quality Assessment

We coded the collected literature that met the inclusion criteria. The coding content mainly extracted data from three aspects: literature characteristics, dependent variables, and moderating variables. The literature characteristic information included the author, publication year, effect size (*k*), and sample size (*N*). In this study, the Pearson correlation coefficient *r* was used as the effect size. The dependent variable of the study was children’s social behavior, which was specifically divided into two types: positive social behavior (PSB) and negative social behavior (NSB). There were seven moderating variables included: children’s gender, children’s age stage, SES, type of father involvement, father involvement reporter and cultural background. Among them, the type of father involvement was explicitly divided into four types: general involvement (GI), supportive involvement (SI), non-coercive control (NC) and negative involvement (NI). Children’s age stage was divided according to the average age of children in included studies: preschool period (3–6 years old) and school-age period (7–12 years old). Cultural background was coded based on the geographic location and dominant cultural context of the study’s participants. Studies conducted in East Asian, Southeast Asian, or South Asian countries were classified as Eastern cultural background (EC), while those in North America, Western Europe, Australia, or New Zealand were classified as Western cultural background (WC). SES was categorized into three levels (High (H)/Medium (M)/Low (L)) based on reported income, parental education, or occupation. For studies that did not explicitly report SES indicators or provided insufficient detail to assign a level, these cases were coded as “not reported (NR)”. The father involvement reporter reflected the person who reported the specific situation of the father involvement in the study, including the parents and children. Child gender was reflected in the proportion of female children reported in each study. When the proportion was not specified, these studies were coded as “not reported (NR)”.

Moreover, methodological quality of the included cross-sectional observational studies was assessed using the Newcastle–Ottawa Scale (NOS) adapted for cross-sectional studies. The adapted NOS includes 8 items across four domains: study aim, participant selection, comparability, and outcome assessment, with a maximum score of 16 points. Referring to the suggestions of the existing meta-analysis studies (Hillen et al., 2017), studies with a total score of 0–8 were of low quality (L), 9–12 were of medium quality (M), and 13–16 were of high quality (H). Among the 37 included studies, 27 were of high quality, 10 were of medium quality, and there were no low-quality studies (The detailed item-level scores for the quality assessment of each study are provided in the Supplementary Materials).

Two independent coders extracted and coded all information. To assess coding reliability, we calculated Cohen’s kappa coefficient, which reached 0.92, indicating consistency. The specific information of the literature is shown in Table 1 (The detailed data for each study are provided in the Supplementary Materials).

### 2.4. Data Analyses

We performed all primary effect and moderator analyses using R (version 4.4.2) with the metafor package (version 3.0), which supports multivariate and multilevel meta-analytic modeling. The Pearson correlation coefficient (*r*) was selected as the effect size. For studies reporting only F, t, *χ*^2^ or *β* values, these statistics were converted to *r* using validated formulas (Card, 2015; Peterson & Brown, 2005).

$$r=\sqrt{\frac{t^{2}}{t^{2}+df}}\;\;\;\;\;\;\;\;\;r=\sqrt{\frac{F}{F+df_{e}}}\;\;\;\;\;\;\;\;\;r=\sqrt{\frac{x^{2}}{x^{2}+N}}\;r=\beta \times 0.98+0.05(\beta \geq 0)\;\;\;\;\;\;\;\;\;\;r=\beta \times 0.98\left(\beta <0\right)$$

To achieve a data distribution that better approximates a normal distribution, begin by applying Fisher’s Z transformation to the correlation coefficient *r*. After completing the analysis, convert the Z value back to the *r* value to make the results easier to interpret. In meta-analysis, the criteria for judging the magnitude of correlation coefficient r as an effect size are: small effect (0.10 ≤ *r* < 0.20), medium effect (0.20 ≤ *r* ≤ 0.30), large effect (*r* > 0.30) (Gignac & Szodorai, 2016).

This study used a three-level meta-analytic model to explicitly account for dependent effect sizes nested within studies, addressing non-independence from multiple correlated estimates per sample. Traditional single-level methods assume independence, requiring one effect size per study to avoid dependency issues (Assink & Wibbelink, 2016). Ignoring such dependencies inflates Type I error rates and overestimates overall effects.

In this model, Level 1 variance represents sampling error of individual effect sizes, Level 2 variance captures variability among effect sizes derived from the same sample, and Level 3 variance reflects between-study heterogeneity (Cheung, 2014). All model parameters were estimated using a mixed-effects framework with restricted maximum likelihood (REML) estimation to ensure robust inference (Christ, 2010).

Heterogeneity was assessed using multiple complementary tests. First, the Q-test was used to evaluate overall heterogeneity in effect sizes, with a significant Q indicating that between-study variation exceeds random sampling error. Next, one-tailed log-likelihood ratio tests were conducted to examine the significance of Level 2 and Level 3 variances. The magnitude of heterogeneity at each level was quantified using the I^2^ statistic: I^2^ < 25% indicates low heterogeneity, I^2^ ≈ 50% indicates moderate heterogeneity, and I^2^ > 75% indicates high heterogeneity (Higgins et al., 2003).

For moderation analyses, we examined both categorical and continuous moderators. When a study reported multiple effect sizes of the father involvement–child social behavior relationship from the same sample (for example, by different types of father involvement), all effect sizes were extracted and analyzed using subgroup analysis for categorical moderators or meta-regression for continuous moderators. Categorical moderators included father involvement type, child age stage, cultural background, SES, and reporter of father involvement. To ensure statistical stability in subgroup analyses, each moderator level required a minimum of three effect sizes. The proportion of females in the sample was treated as a continuous moderator and analyzed via meta-regression. To evaluate the robustness of the primary results, a sensitivity analysis was conducted using the leave-one-out method. Each study was sequentially removed from the dataset, and the overall effect size was re-estimated to assess whether any single study disproportionately influenced the pooled result.

Publication bias was assessed using three complementary methods: funnel plots, Rosenthal’s Fail-Safe Number and Egger’s regression test. Publication bias referred to the fact that studies with significant results were more likely to be accepted and published, making it difficult to obtain studies with non-significant results during the literature search process. This could affect the accuracy of meta-analysis results (Rothstein et al., 2005). In terms of the funnel plot, when the effect results were all at the top of the funnel plot and were relatively evenly distributed on both sides of the average effect size, it indicated that the meta-analysis was less likely to be affected by publication bias (Rothstein, 2008). The Fail-Safe Number was an important indicator in meta-analysis for evaluating publication bias and the robustness of results; it was generally believed that when Fail-Safe Number > 5*k* + 10 (*k* represents the number of effect sizes) (Rosenthal, 1979), the impact of publication bias on the current meta-analysis result could be ignored. Egger’s regression test detects publication bias by examining the linear relationship between each study’s effect size and its standard error. A non-significant result (*p* > 0.05) for Egger’s regression was interpreted as indicating no significant publication bias.

## 3. Results

We finally obtained 37 valid studies, and a total of 249 effect sizes were extracted. Among them, there were 111 effect sizes for the relationship between father involvement and children’s positive social behaviors, and 138 for the relationship between father involvement and children’s negative social behaviors. These studies were all published between 2010 and 2025. The sample size ranged from 24 to 5064, with an average sample size of N = 397.

### 3.1. Publication Bias and Sensitivity Analysis

**Figure 2:**
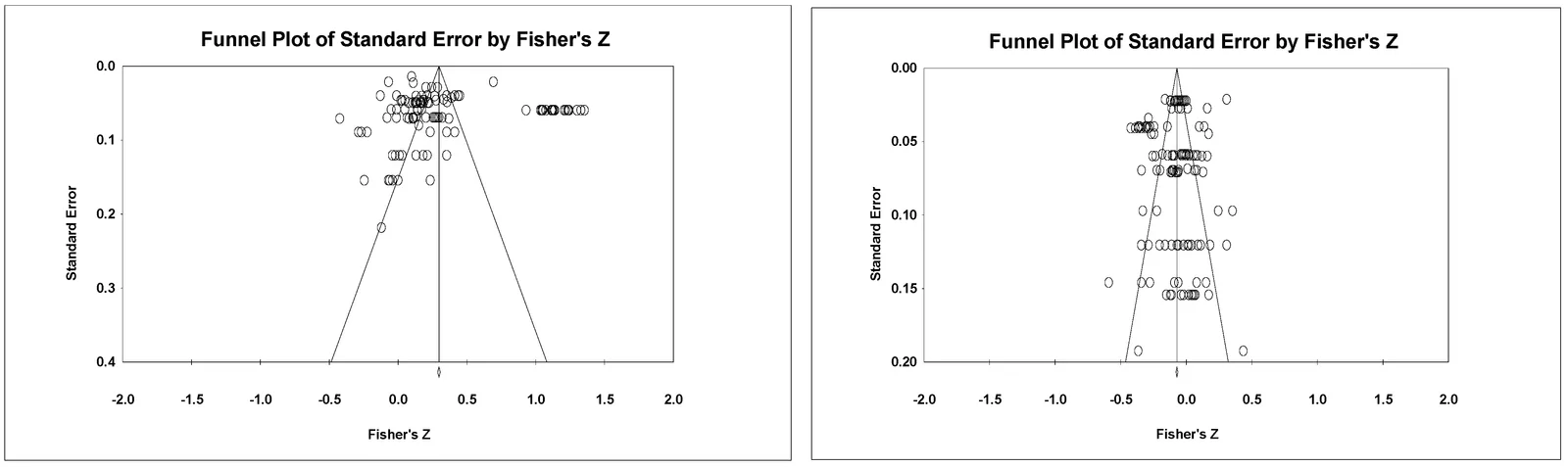
Funnel plot for the relationship between FI and PSB, NSB.

Figure 2 shows the distribution of the meta-analysis effect sizes of the relationships between FI and children’s positive social behaviors, as well as father involvement and children’s negative social behaviors. Some of the effect size results in the two figures are not at the top of the funnel plot and are not evenly distributed on both sides of the average effect size, indicating that there may be publication bias in the results of this meta-analysis. Therefore, this study supplemented the publication bias assessment using both the fail-safe N and Egger’s test. The Fail-Safe Number values for the relationships are 7622 and 4003, respectively, both exceeding the threshold of 5*k* + 10 proposed by Rosenthal (1979). Additionally, Egger’s test indicated no significant asymmetry in the distribution of effect sizes for either positive social behavior (intercept = 0.324, SE = 0.099, *p* = 0.154 > 0.05) or negative social behavior (intercept = −0.109, SE = 0.045 *p* = 0.562 > 0.05). Together, these results suggest that the findings of this meta-analysis are unlikely to be influenced by publication bias, supporting the reliability and robustness of the conclusions.

This study performed a leave-one-out sensitivity analysis to verify the stability of the meta-analytic results for the associations between FI and children’s PSB, as well as between FI and children’s NSB. The results showed that after sequentially excluding each individual independent study, the 95% confidence intervals (CIs) of the pooled effect sizes for both meta-analyses did not cross zero, with only minimal fluctuation in the effect size values. This indicates that the meta-analytic results of the present study have good stability and robustness, and are not affected by the extreme influence of any single independent study.

### 3.2. Primary Meta-Analysis

This study adopted a three-level meta-analytic method to systematically examine the heterogeneity structure and statistical characteristics of the associations between FI and children’s PSB and NSB. Model fitting results based on restricted maximum likelihood estimation (REML) revealed that FI was significantly positively associated with children’s PSB (*r* = 0.203, 95% CI [0.100, 0.307], *p* < 0.001) and significantly negatively associated with children’s NSB (*r* = −0.087, 95% CI [−0.135, −0.038], *p* < 0.001). Heterogeneity analyses via the three-level model indicated that for children’s PSB, the within-study true effect size variance was σ^2^_2_ = 0.0171 (Level 2, I^2^_2_ = 75.16%) and the between-study true effect size heterogeneity was σ^2^_3_ = 0.0589 (Level 3, I^2^_3_ = 21.81%), with high heterogeneity predominantly at Level 2, suggesting that within-study variations in effect sizes may stem from differences in the dimensions of FI and measures of children’s PSB. For children’s NSB, the within-study true effect size variance was σ^2^_2_ = 0.0092 (Level 2, I^2^_2_ = 48.53%) and the between-study true effect size heterogeneity was σ^2^_3_ = 0.0101 (Level 3, I^2^_3_ = 44.13%), with moderate heterogeneity at both Level 2 and Level 3, indicating coexisting within-study associative variations and between-study true effect differences, which may relate to variations in the measurement of FI, assessment tools for children’s NSB, and research designs. The heterogeneity of both associations was not merely attributable to sampling error. Accordingly, moderation analyses were further conducted in this study to explore the potential sources of heterogeneity in depth.

### 3.3. Moderation Effect

#### 3.3.1. The Moderating Effect of the Relationship Between FI and PSB

We conducted moderation effect analyses on the association between FI and children’s PSB. As shown in Table 2, the results revealed that cultural background exerted a significant moderating effect (QM = 23.830, *p* < 0.001), with the correlation between FI and children’s PSB being significantly stronger in Eastern cultural backgrounds than in Western cultural backgrounds. Children’s age stage also had a significant moderating effect (QM = 14.389, *p* < 0.001): the association between FI and children’s PSB was significant for preschool children aged 3–6 years, while it was only marginally significant for school-age children aged 7–12 years. SES exerted a significant moderating effect (QM = 11.740, *p* < 0.01): the correlation between FI and children’s PSB was the highest in high-SES families, followed by middle-SES families, and did not reach statistical significance in low-SES families. The type of FI exerted a significant moderating effect (QM = 59.864, *p* < 0.001). Specifically, the correlation between FI and children’s PSB was significantly positive for type GI, NC and SI, while it was significantly negative for type NI. The reporter of FI presented a significant moderating effect (QM = 15.786, *p* < 0.01): only paternal self-reporting was significantly positively correlated with children’s PSB, whereas the correlation did not reach statistical significance in samples with child-reported, mother-reported, or parent-jointly reported data. Study quality significantly moderated the FI and children’s PSB association (QM = 15.743, *p* < 0.001). The positive association was significant in both moderate- and high-quality studies, with a stronger effect in moderate-quality research.

In addition, we examined the moderating effect of the proportion of females in the child sample on the association between FI and children’s PSB. The results showed that the correlation between FI and children’s PSB did not change significantly with the increase in the proportion of females in the child sample (*r* = 0.003, 95% CI [−0.007, 0.013], *p* = 0.593).

#### 3.3.2. The Moderating Effect of the Relationship Between FI and NSB

We conducted moderation effect analyses on the association between FI and children’s NSB. As shown in Table 3, the results revealed that children’s age stage also had a significant moderating effect (QM = 11.667, *p* < 0.01): the negative association between FI and children’s NSB was significant for preschool children aged 3–6 years, and also reached statistical significance for school-age children aged 7–12 years, with a slightly stronger effect size observed in school-age children. SES exerted a significant moderating effect (QM = 10.581, *p* < 0.05): the negative association between FI and children’s NSB was the highest in high-SES families, followed by middle-SES families, and did not reach statistical significance in low-SES families. The type of father involvement exerted a significant moderating effect (QM = 124.809, *p* < 0.001). Specifically, the correlation between FI and children’s NSB was significantly negative for type GI, NC and SI, while it was significantly positive for type NI. The reporter of FI presented a significant moderating effect (QM = 13.583, *p* < 0.01): only paternal self-reporting was significantly negatively correlated with children’s NSB, whereas the correlation did not reach statistical significance in samples with child-reported, mother-reported, or parent-jointly reported data. Cultural background exerted a significant moderating effect (QM = 39.901, *p* < 0.001), with the negative association between FI and children’s NSB being significantly stronger in Eastern cultural backgrounds than in Western cultural backgrounds. Study quality significantly moderated the FI and children’s NSB association (QM = 13.336, *p* < 0.001). The negative association was significant in both moderate- and high-quality studies, with a stronger effect in moderate-quality research.

In addition, we examined the moderating effect of the proportion of females in the child sample on the association between FI and children’s NSB. The results showed that the correlation between FI and children’s NSB did not change significantly with the increase in the proportion of females in the child sample (*r* = −0.0013, 95% CI [−0.0046, 0.0020], *p* = 0.4326).

## 4. Discussion

The current study employed a three-level meta-analytic model, synthesizing 249 effects from 37 studies, systematically examining the associations between father involvement and children’s social behaviors (both positive and negative), and addressing the research gap in understudied children’s negative behavior (e.g., aggression, withdrawal).

Our findings indicate a moderate positive association between father involvement and children’s positive social behavior (*r* = 0.203, 95% CI [0.100, 0.307], *p* < 0.001), consistent with prior studies (Khawaja et al., 2024; Pekel-Uludağlı, 2024; van der Storm et al., 2022; I. Y. Wang & Cheung, 2023). Father involvement is more than just economic provision, but also serves as a potential behavioral role model. Fathers may offer unique forms of care and support that correlate with children’s social development, distinct from patterns often associated with maternal care (Lamb, 1975).

A low negative association was observed between father involvement and children’s negative social behavior (*r* = −0.087, 95% CI [−0.135, −0.038], *p* < 0.001), aligning with prior research (Caldwell et al., 2010; Fagan & Wildfeuer, 2022; Levant et al., 2014). Father involvement may correlate with greater rule awareness in children through activities such as rule-setting and behavioral guidance, which has been hypothesized to link to lower levels of aggressive or destructive behavior. Additionally, the paternal role differs from the maternal role in its focus on introducing children to the external world and reality (Lamb, 1975), patterns that may correlate with greater confidence and social interaction skills.

This study also examined seven moderators in this study. By age stage, children’s developmental period significantly moderated the association between FI and children’s PSB. Subgroup analyses revealed that the correlation between FI and PSB was statistically significant only among preschool children aged 3–6 years, while it reached only marginal significance for school-age children aged 7–12 years. This pattern suggests that FI may play a more salient role in fostering positive social behavior during the preschool years, when children’s social development is highly malleable and heavily shaped by primary caregivers such as fathers (Santos et al., 2022). Notably, the protective effect of FI against children’s NSB remained stable across developmental stages. For both preschoolers and school-age children, FI was significantly negatively associated with NSB, with comparable effect sizes. This finding aligns with prior research (H. C. Chen et al., 2004), indicating that FI exerts a consistent buffering effect on children’s NSB throughout childhood.

With regard to SES, fathers in middle and high-SES families showed stronger associations with children’s social behavior than those in low-SES families. This may relate to disparities in resource investment and parenting styles: middle/high-SES resources amplify the positive effects of father involvement, whereas stressors in low-SES contexts may hinder prosocial development (Ward & Lee, 2020).

Regarding FI types, GI, SI and NC involvement were all significantly associated with higher levels of children’s PSB and lower levels of NSB, while NI showed the opposite pattern. Specifically, NC exhibited the strongest correlation with PSB and NSB, followed by GI and SI. Prior work has linked general involvement (e.g., basic caregiving and playtime) to prosocial habits through consistent daily support and routine interactions (Dubeau et al., 2013), while supportive involvement (e.g., emotional encouragement and cognitive guidance) has been associated with greater social confidence and cooperative behavior by fostering secure attachment and socioemotional competence (Semiz & Ören, 2024). These findings suggest that emotional connection and responsive, consistent support are key correlates of the positive links between father involvement and children’s social behavior. Notably, NC, characterized by warm, rule-guided discipline and supervision, emerged as the most impactful form of father involvement across both outcomes. Beyond its established role in mitigating negative behaviors by teaching children to resolve conflicts through communication rather than aggression (St George et al., 2017; J. Zhang et al., 2021), our results show it also strongly correlates with higher positive social behavior, likely by providing clear boundaries that help children internalize prosocial norms and build self-regulation skills.

In contrast, negative involvement (e.g., physical punishment, harsh criticism, or emotional neglect) emerged as a significant risk factor. As our analyses confirmed, NI was associated with lower PSB and higher NSB, aligning with prior research documenting the detrimental effects of harsh or disengaged fathering on children’s social adjustment (Carrasco et al., 2009; H. C. Chen et al., 2004). Collectively, these patterns demonstrate that the quality and specific types of father involvement are critically important for children’s social development.

From the perspective of reporters of father involvement, only paternal self-reporting was significantly associated with both children’s PSB and NSB. In contrast, child-reported, mother-reported, and parent-jointly reported father involvement showed no significant correlations with either outcome, with only child-reported involvement marginally linked to PSB. This reveals systematic reporting bias in perceptions of father involvement, wherein fathers tend to report more positive involvement and its benefits, while other family members may hold different views (Charles et al., 2018; Dyer et al., 2014). Future research should integrate multiple reporter perspectives to improve the rigor and internal validity of findings.

Culturally, the associations between FI and children’s social behaviors were significantly moderated by cultural background. Specifically, FI was strongly linked to higher levels of children’s PSB in Eastern cultural contexts but not in Western contexts. For children’s NSB, the protective effect of FI was also much stronger in Eastern cultures than in Western cultures. This pattern likely aligns with Eastern cultural values that emphasize social norms, moral principles, and cooperative harmony in childrearing, which may amplify both the positive role of FI in fostering prosocial development and its buffering effect against behavioral problems. In contrast, Western contexts, which tend to prioritize individual autonomy, may dilute such culturally framed paternal influences.

Study quality significantly moderated the associations between father involvement and children’s social behaviors. Moderate-quality studies showed stronger correlations for both PSB and NSB than high-quality studies, indicating that less rigorous designs may inflate effect sizes, while high-quality research yields more conservative and reliable estimates.

Additionally, children’s gender did not significantly moderate the relationship between FI and children’s PSB or NSB. This finding diverges from prior research, which reported significant gender differences; father positivity and corporal punishment were significantly associated with girls’ social behavior but not boys’ (Gryczkowski et al., 2018).

In conclusion, FI is consistently associated with children’s social development, in both positive and negative directions. This meta-analysis highlights the association between FI and children’s social behaviors (PSB/NSB), systematically examining multilevel moderators across child, family and societal domains. This transcends prior single-dimensional analyses and lays the groundwork for cross-cultural theoretical frameworks of father involvement.

## 5. Conclusions

The present meta-analysis synthesized 37 studies with 249 effect sizes to systematically examine the associations between father involvement and children’s positive and negative social behavior, as well as the moderating roles of child, family, and cultural factors. The core findings revealed a moderate positive association between father involvement and children’s positive social behavior, and a weak negative association between father involvement and children’s negative social behavior. Further moderation analyses confirmed that the type of father involvement, children’s age stage, family socioeconomic status, reporter of father involvement, cultural background and study quality collectively moderated these associations.

These findings advance the understanding of father involvement by verifying its differential links to children’s dual-dimensional social behavior. Nevertheless, this study has several limitations: (1) most included studies have been cross-sectional, lacking trajectory tracking. Longitudinal designs are needed to address this gap; (2) given the high level of heterogeneity, our subgroup and meta-regression analyses explained a portion of the observed heterogeneity. This study examined moderators like age, gender, and SES, future research could incorporate additional child characteristics (temperament, only child status) and measurement approaches (questionnaire/observation); (3) age categorization into two groups due to sample heterogeneity may obscure stage-specific differences; continuous variable analysis is recommended; (4) focusing solely on father involvement, future studies could compare differential effects of father and mother involvement.

## Figures and Tables

**Table 1:** Studies included in the meta-analysis.

Author	*k*	N	Percent Female	Age Group	Culture	Type of FI	Social Behavior	SES	Reporter	Study Quality
(Amaral et al., 2019)	5	283	50.90%	3–6	WC	GI/NC/NI	PSB/NSB	NR	Parent	H
(Bagán et al., 2019)	6	635	46.30%	3–6	WC	SI/NI	PSB/NSB	L	Children	H
(Caldwell et al., 2010)	4	287	0%	7–12	WC	GI/SI	NSB	L	Father/Children	H
(Caldwell et al., 2014)	6	287	0%	7–12	WC	GI/NC	NSB	L	Father	H
(X. P. Chen & An, 2019)	1	432	52.30%	7–12	EC	SI	PSB	M	Children	H
(Dubeau et al., 2013)	18	45	44.40%	3–6	WC	GI/SI/NC	PSB/NSB	H	Father	M
(Fagan & Wildfeuer, 2022)	3	1988	49.70%	7–12	WC	GI	PSB/NSB	L	Father	H
(Fan & Wang, 2019)	15	210	NR	7–12	EC	GI/SI/NC	PSB/NSB	H	Father	M
(Gao et al., 2020)	6	604	42.90%	7–12	EC	GI/SI/NC	NSB	H	Father	H
(Gomes et al., 2013)	7	50	42%	3–6	WC	GI/SI/NC	NSB	H	Father	M
(Gryczkowski et al., 2018)	5	129	49.60%	7–12	WC	GI/SI/NC/NI	PSB	M	Parent	M
(Jaffee et al., 2003)	3	2232	51%	3–6	WC	GI/NI	NSB	NR	Mother	H
(Kennedy et al., 2015)	2	30	43.30%	3–6	WC	GI/NI	NSB	H	Mother	M
(Khawaja et al., 2024)	1	160	42.20%	7–12	EC	GI	PSB	M	Father	M
(Levant et al., 2014)	1	50	68%	3–6	WC	GI	NSB	H	Mother	M
(Q. Liu & Lu, 2022)	20	286	NR	3–6	EC	GI/SI	PSB	H	Father	M
(R. Y. Liu, 2022)	15	417	49.60%	3–6	EC	GI/SI/NC	PSB	M	Father	M
(X. J. Liu et al., 2022)	1	130	47%	3–6	EC	SI	PSB	H	Father	H
(Nóblega et al., 2024)	10	203	57.63%	3–6	WC	GI/NC/NI	PSB/NSB	M	Mother	H
(Paquette et al., 2023)	24	72	44.40%	7–12	WC	GI/NI	PSB/NSB	H	Father	H
(Pekel-Uludağlı, 2024)	2	216	49.10%	3–6	WC	GI	PSB/NSB	M	Mother	H
(Ramchandani et al., 2010)	1	5064	48.90%	3–6	WC	GI	PSB	NR	Mother	H
(Ren & Zhang, 2018)	4	109	59%	3–6	EC	SI/NI	NSB	H	Father	M
(Semiz & Ören, 2024)	4	504	50.20%	3–6	WC	GI/NI	PSB/NSB	NR	Father	H
(St George et al., 2017)	1	24	29%	3–6	WC	GI	PSB	H	Father	H
(Torres et al., 2014)	15	295	52%	3–6	WC	GI/SI/NC	PSB/NSB	M	Parent	H
(I. Y. Wang & Cheung, 2023)	9	211	46.90%	3–6	EC	GI/SI/NC	PSB	M	Father	H
(X. Wang et al., 2019)	1	2016	48.60%	7–12	WC	GI	NSB	L	Mother	H
(J. Wang et al., 2023)	4	1231	48.10%	7–12	EC	SI	PSB	H	Mother	H
(S. M. Wang et al., 2024)	2	2253	46.70%	3–6	EC	SI/NI	PSB	M	Father	H
(W. Wu et al., 2023)	4	613	34.70%	7–12	EC	GI/SI/NC	PSB	L	Children	H
(M. Wu et al., 2024)	1	588	52.89%	7–12	EC	SI	PSB	L	Children	H
(Xia & He, 2023)	1	864	NR	3–6	EC	GI	NSB	L	Father	H
(P. Yang et al., 2024)	6	1349	48%	7–12	WC	GI/SI/NI	NSB	L	Father	H
(Yoon et al., 2025)	22	2040	50.34%	3–6	WC	GI/SI	NSB	L	Parent	H
(J. Z. Zhang et al., 2022)	7	478	47.50%	3–6	EC	GI/SI/NC	PSB	H	Father	H
(Y. F. Zhang et al., 2025)	12	635	50.87%	3–6	EC	GI/SI/NC	PSB/NSB	M	Father	H

Note: *k* number of effect sizes; N sample size; FI father involvement; GI general involvement; SI supportive involvement; NC non-coercive; NI negative involvement; EC Eastern culture; WC Western culture; PSB positive social behavior; NSB negative social behavior; H high; M medium; L low; NR no report.

**Table 2:** Moderating variable effects on the relationship between FI and PSB.

Moderator	Subgroup	*k*	*r*	95%CI	*p*	QM
Age stage	3–6	86	0.208	[0.084, 0.326]	0.001	14.389 ***
	7–12	25	0.183	[−0.001, 0.356]	0.051	
SES	H	52	0.244	[0.052, 0.418]	0.013	11.740 **
	M	47	0.181	[0.009, 0.343]	0.040	
	L	9	0.167	[−0.111, 0.421]	0.238	
Type of FI	GI	53	0.217	[0.118, 0.313]	<0.001	59.864 ***
	SI	34	0.206	[0.094, 0.313]	<0.001	
	NC	17	0.256	[−0.289, −0.003]	<0.001	
	NI	7	−0.149	[0.156, 0.352]	0.045	
Reporter	Father	81	0.244	[0.108, 0.372]	0.001	15.786 **
	Mother	10	0.128	[−0.136, 0.375]	0.342	
	Parent	11	0.061	[−0.236, 0.348]	0.690	
	Children	9	0.220	[−0.042, 0.454]	0.099	
Culture	EC	74	0.304	[0.183, 0.416]	<0.001	23.830 ***
	WC	37	0.075	[−0.065, 0.212]	0.293	
Study quality	H	64	0.176	[0.062, 0.286]	0.003	15.743 ***
	M	47	0.289	[0.073, 0.479]	0.009	

Note: *k* number of effect sizes; FI father involvement; GI general involvement; SI supportive involvement; NC non-coercive; NI negative involvement; EC Eastern culture; WC Western culture; PSB positive social behavior; H High; M Medium; L Low; QM between-group heterogeneity Q statistic; ** *p* < 0.01, *** *p* < 0.001.

**Table 3:** Moderating variable effects on the relationship between FI and NSB.

Moderator	Subgroup	*k*	*r*	95%CI	*p*	QM
Age stage	3–6	97	−0.083	[−0.143, −0.022]	0.007	11.667 **
	7–12	41	−0.094	[−0.179, −0.007]	0.034	
SES	H	58	−0.119	[−0.214, −0.023]	0.016	10.581 *
	M	45	−0.106	[−0.231, 0.022]	0.105	
	L	25	−0.067	[−0.156, 0.023]	0.147	
Type of FI	GI	53	−0.119	[−0.165, −0.074]	<0.001	124.809 ***
	SI	34	−0.128	[−0.179, −0.077]	<0.001	
	NC	17	−0.148	[−0.206, −0.089]	<0.001	
	NI	7	0.161	[0.098, 0.224]	<0.001	
Reporter	Father	83	−0.108	[−0.170, −0.046]	0.001	15.786 **
	Mother	14	−0.073	[−0.185, 0.040]	0.206	
	Parent	36	−0.043	[−0.183, 0.120]	0.680	
	Children	5	−0.032	[−0.165, 0.080]	0.496	
Culture	EC	29	−0.213	[−0.281, −0.144]	<0.001	39.901 ***
	WC	209	−0.044	[−0.084, −0.004]	0.030	
Study quality	H	106	−0.072	[−0.127, −0.018]	0.010	15.743 ***
	M	32	−0.136	[−0.236, −0.033]	0.010	

Note: *k* number of effect sizes; FI father involvement; GI general involvement; SI supportive involvement; NC non-coercive; NI negative involvement; EC Eastern culture; WC Western culture; NSB negative social behavior; H High; M Medium; L Low; QM between-group heterogeneity Q statistic; * *p* < 0.05, ** *p* < 0.01, *** *p* < 0.001.

## Data Availability

The extracted effect size dataset is available from the corresponding author upon request.

## Supplementary Materials

The following supporting information can be downloaded at: https://www.mdpi.com/article/10.3390/bs16081426/s1, Table S1: Item-Level Newcastle-Ottawa Scale (NOS) Scores for Included Studies; Table S2: Effect sizes with corresponding study and sample characteristics; Database-Specific Search Strategies.

## Abbreviations

The following abbreviations are used in this manuscript:

SES	Socioeconomic Status
FI	Father Involvement
PSB	Positive Social Behavior
NSB	Negative Social Behavior
GI	General Involvement
SI	Supportive Involvement
NC	Non-coercive
NI	Negative Involvement
EC	East Culture
WC	West Culture
