# Supplementary material for: Father Involvement and Children’s Social Behavior: A Three-Level Meta-Analysis
Source: Behav Sci (Basel). 2026 Aug 19;16(8):1426. doi: 10.3390/bs16081426 (PMC13509281; doi:10.3390/bs16081426)
Supplement: Supplementary file 1 [file behavsci-16-01426-s001.zip › behavsci-4205966-supplementary.pdf]

Table S1. Item-Level Newcastle-Ottawa Scale (NOS) Scores for Included Studies

Studies	Clearness of Stated Aim (0–2)	Sample Selection				Comparability		Outcome		NOS Total Score (0–16)
		Sample Representativeness (0–2)	Sample Size (0–2)	Non-Respondents (0–2)	Exposure Assessment (0–2)	Control of Confounding Factors (0–1)	Comparability of Participants (0–1)	Assessment of the Outcome (0–2)	Statistical Tests (0–2)	
Amaral et al., 2019	2	1	2	1	2	1	1	2	2	14
Bagán et al., 2019	2	1	2	1	2	1	1	2	2	14
Caldwell et al., 2010	2	1	2	2	2	1	1	2	2	15
Caldwell et al., 2014	2	1	2	2	2	1	1	2	2	15
Chen & An，2019	2	1	2	2	2	1	1	2	2	15
Dubeau et al., 2013	2	0	0	1	2	0	1	2	1	9
Fagan & Wildfeuer, 2022	2	2	2	2	2	1	1	2	2	16
Fan & Wang, 2019	2	0	1	1	2	0	0	2	1	9
Gao et al., 2020	2	1	2	1	2	1	1	2	2	14
Gomes et al., 2013	2	1	1	0	2	0	0	2	2	10
Gryczkowski et al., 2018	2	0	0	0	2	1	1	2	2	10
Jaffee et al., 2003	2	2	2	2	2	1	1	2	2	16
Kennedy et al., 2015	2	0	0	0	2	1	1	2	1	9
Khawaja et al., 2024	2	0	0	2	2	1	1	2	2	12
Levant et al., 2014	2	0	0	0	2	1	1	2	1	9
Liu & Lu, 2022	2	0	1	1	2	0	0	2	1	9
Liu, 2022	2	1	1	1	2	1	1	2	2	12
Liu et al., 2022	2	1	1	1	2	1	1	2	2	13
Nóblega et al., 2024	2	1	1	1	2	1	1	2	2	13
Paquette et al., 2023	2	1	2	1	2	1	1	2	2	14
Pekel-Uludağlı, 2024	2	2	2	1	2	1	1	2	2	15
Ramchandani et al., 2010	2	2	2	1	2	1	1	2	2	15
Ren & Zhang, 2018	2	1	1	0	2	0	0	2	2	10
Semiz & Ören, 2024	2	1	2	1	2	1	1	2	2	14
St George et al., 2017	2	2	2	1	1	1	1	2	1	13
Torres et al., 2014	2	1	2	1	2	1	1	2	2	14
Wang & Cheung, 2023	2	1	2	1	2	1	1	2	2	14

Studies	Clearness of Stated Aim (0–2)	Sample Selection				Comparability		Outcome		NOS Total Score (0–16)
		Sample Representativeness (0–2)	Sample Size (0–2)	Non-Respondents (0–2)	Exposure Assessment (0–2)	Control of Confounding Factors (0–1)	Comparability of Participants (0–1)	Assessment of the Outcome (0–2)	Statistical Tests (0–2)	
Wang et al., 2019	2	2	2	2	2	1	1	2	2	16
Wang et al., 2023	2	1	2	2	2	1	1	2	2	15
Wang et al., 2024	2	2	2	2	2	1	1	2	2	16
Wu et al., 2023	2	1	2	1	2	1	1	2	2	14
Wu et al., 2024	2	1	2	2	2	1	1	2	2	15
Xia & He, 2023	2	1	2	1	2	1	1	2	2	14
Yang et al., 2024	2	1	2	2	2	1	1	2	2	15
Yoon et al., 2025	2	2	2	2	2	1	1	2	2	16
Zhang et al., 2022	2	1	2	2	2	1	1	2	2	15
Zhang et al., 2025	2	1	2	1	2	1	1	2	2	14

Table S2. Effect sizes with corresponding study and sample characteristics

author	*k*	N	*r*	type	SB	Age groupe	Gulture	SES	Report	Assessment	Female
Amaral et al., 2019	5	283	-0.23	GI	NSB	3~6	W	NR	Parent	H	50.90%
			0.12	GI	NSB						
			0.14	NC	PSB						
			-0.25	NC	NSB						
			0.16	NI	NSB						
Bagán et al., 2019	6	635	0.208	SI	PSB	3~6	W	L	Children	H	46.30%
			-0.142	SI	NSB						
			-0.128	NI	PSB						
			-0.01	NI	PSB						
			0.136	NI	NSB						
			0.099	NI	NSB						
Caldwell et al., 2010	4	287	-0.1	GI	NSB	7~12	W	L	Father	H	0%
			0	GI	NSB				Children		
			-0.09	SI	NSB				Father		
			-0.14	SI	NSB				Children		
Caldwell et al., 2014	6	287	0.086	GI	NSB	7~12	W	L	Father	H	0%
			0.073	GI	NSB						
			0.055	NC	NSB						
			0.023	NC	NSB						
			-0.034	NC	NSB						
			-0.108	NC	NSB						
Chen & An., 2019	1	432	0.342	SI	PSB	7~12	E	M	Children	H	52.30%

Dubeau et al., 2013	18	45	-0.24	GI	PSB	3~6	W	H	Father	M	44.40%
			0.23	GI	PSB						
			-0.04	GI	PSB						
			0	GI	PSB						
			0.05	GI	NSB						
			-0.04	GI	NSB						
			-0.11	GI	NSB						
			-0.04	GI	NSB						
			-0.11	GI	NSB						
			0.17	GI	NSB						
			0.06	GI	NSB						
			0.07	GI	NSB						
			-0.07	SI	PSB						
			0.02	SI	NSB						
			-0.12	SI	NSB						
			-0.06	NC	PSB						
			-0.02	NC	NSB						
			-0.15	NC	NSB						
Fagan & Wildfeuer., 2022	3	1988	0.11	GI	PSB	7~12	W	L	Father	H	49.70%
			-0.12	GI	NSB						
			-0.09	GI	NSB						
Fan & Wang, 2019	15	210	-0.012	GI	PSB	3~6	E	H	Father	H	NR
			-0.196	GI	NSB						
			0.078	GI	NSB						
			0.114	SI	PSB						

			-0.079	SI	PSB						
			-0.328	SI	NSB						
			0.063	SI	NSB						
			-0.093	SI	NSB						
			-0.058	SI	NSB						
			0.066	NC	PSB						
			0.106	NC	PSB						
			-0.22	NC	NSB						
			-0.107	NC	NSB						
			-0.102	NC	NSB						
			-0.103	NC	NSB						
Gao et al., 2020	6	604	-0.37	GI	NSB	7~12	E	H	Father	M	42.90%
			-0.4	GI	NSB						
			-0.28	SI	NSB						
			-0.3	SI	NSB						
			-0.33	NC	NSB						
			-0.35	NC	NSB						
Gomes et al., 2013	7	50	0.08	GI	NSB	3~6	W	H	Father	M	42%
			-0.33	GI	NSB						
			0.08	GI	NSB						
			-0.27	GI	NSB						
			-0.09	SI	NSB						
			-0.06	SI	NSB						
			0.15	NC	NSB						
Gryczkowski et al., 2018	5	129	0.39	GI	PSB	7~12	W	M	Parent	M	49.60%

			0.34	SI	PSB						
			-0.22	NC	PSB						
			-0.28	NC	PSB						
			-0.26	NI	PSB						
Jaffee et al., 2003	3	2232	-0.16	GI	NSB	3~6	W	NR	Mother	H	51%
			-0.16	GI	NSB						
			0.3	NI	NSB						
Kennedy et al., 2015	2	30	-0.35	GI	NSB	3~6	W	H	Mother	M	43.30%
			0.41	NI	NSB						
Khawaja et al., 2024	1	160	0.15	GI	PSB	7~12	E	M	Father	M	42.20%
Levant et al., 2014	1	50	-0.53	GI	NSB	3~6	W	H	Mother	M	68%
Liu & Lu, 2022	20	286	0.809	GI	PSB	3~6	E	H	Father	M	NR
			0.791	GI	PSB						
			0.839	GI	PSB						
			0.846	GI	PSB						
			0.814	GI	PSB						
			0.862	GI	PSB						
			0.812	GI	PSB						
			0.731	GI	PSB						
			0.781	GI	PSB						
			0.875	GI	PSB						
			0.807	GI	PSB						
			0.869	GI	PSB						
			0.845	GI	PSB						
			0.779	GI	PSB						

			0.836	GI	PSB						
			0.783	SI	PSB						
			0.806	SI	PSB						
			0.776	SI	PSB						
			0.843	SI	PSB						
			0.807	SI	PSB						
Liu, 2022	15	417	-0.183	GI	PSB	3~6	E	M	Father	M	49.60%
			0.128	GI	PSB						
			0.108	GI	PSB						
			0.167	GI	PSB						
			0.136	GI	PSB						
			0.223	SI	PSB						
			0.124	SI	PSB						
			0.078	SI	PSB						
			0.139	SI	PSB						
			0.138	SI	PSB						
			0.213	NC	PSB						
			0.163	NC	PSB						
			0.126	NC	PSB						
			0.162	NC	PSB						
			0.160	NC	PSB						
Liu et al., 2022	1	130	0.23	SI	PSB	3~6	E	H	Father	H	47%
Nóblega et al., 2025	10	203	0.354	GI	PSB	3~6	W	M	Mother	H	57.63%
			0.109	GI	PSB						
			-0.119	GI	NSB						

			0.128	GI	NSB						
			-0.08	GI	NSB						
			-0.109	GI	NSB						
			0.08	NC	PSB						
			-0.06	NC	NSB						
			-0.07	NC	NSB						
			-0.4	NI	PSB						
Paquette et al., 2023	24	72	0.18	GI	PSB	7~12	W	H	Father	H	44.40%
			-0.02	GI	PSB						
			0.21	GI	PSB						
			0.03	GI	PSB						
			0.34	GI	PSB						
			0.01	GI	PSB						
			-0.11	GI	NSB						
			-0.07	GI	NSB						
			0.01	GI	NSB						
			0.30	GI	NSB						
			0.11	GI	NSB						
			-0.33	GI	NSB						
			-0.2	GI	NSB						
			-0.06	GI	NSB						
			-0.02	GI	NSB						
			-0.16	GI	NSB						
			-0.28	GI	NSB						
			0.09	GI	NSB						

			-0.04	NI	PSB						
			0.13	NI	PSB						
			0.02	NI	NSB						
			0.01	NI	NSB						
			0.18	NI	NSB						
			0.04	NI	NSB						
Pekel-Uludağlı, 2024	2	216	0.13	GI	PSB	3~6	W	M	Mother	H	49.10%
			0.01	GI	NSB						
Ramchandani et al., 2010	1	5064	0.099	GI	PSB	3~6	W	NR	Mother	H	48.90%
Ren ＆ Zhang, 2018	4	109	-0.32	SI	NSB	3~6	E	H	Father	M	59%
			-0.22	SI	NSB						
			0.34	NI	NSB						
			0.24	NI	NSB						
SEMİZ ＆ ÖREN, 2024	4	504	0.32	GI	PSB	3~6	W	NR	Father	H	50.20%
			-0.26	GI	NSB						
			-0.24	GI	NSB						
			0.169	NI	NSB						
St George et al., 2017	1	24	-0.12	GI	PSB	3~6	W	H	Father	H	29%
Torres et al., 2014	15	295	-0.01	GI	PSB	3~6	W	M	Parent	H	52%
			0.14	GI	PSB						
			-0.05	GI	PSB						
			0.02	GI	NSB						
			-0.04	GI	NSB						
			-0.01	GI	NSB						

			-0.03	GI	NSB						
			-0.04	GI	NSB						
			-0.01	GI	NSB						
			0.17	SI	PSB						
			-0.18	SI	NSB						
			0.00	SI	NSB						
			0.05	NC	PSB						
			-0.02	NC	NSB						
			-0.03	NC	NSB						
Wang & Cheung, 2023	9	211	0.20	GI	PSB	3~6	E	M	Father	H	46.90%
			0.29	GI	PSB						
			0.27	GI	PSB						
			0.20	GI	PSB						
			0.25	GI	PSB						
			0.26	SI	PSB						
			0.27	SI	PSB						
			0.31	SI	PSB						
			0.28	NC	PSB						
Wang et al., 2019	1	2016	-0.089	GI	NSB	7~12	W	L	Mother	H	48.60%
Wang et al., 2023	4	1231	0.2	SI	PSB	7~12	E	H	Mother	H	48.10%
			0.24	SI	PSB						
			0.28	SI	PSB						
			0.24	SI	PSB						
Wang et al., 2024	2	2253	0.6	SI	PSB	3~6	E	M	Father	H	46.70%
			-0.069	NI	PSB						

Wu et al., 2023	4	613	0.13	GI	PSB	7~12	E	L	Children	H	34.70%
			0.13	GI	PSB						
			0.17	SI	PSB						
			0.26	NC	PSB						
Wu et al., 2024	1	588	0.37	SI	PSB	7~12	E	L	Children	H	52.89%
Xia & He, 2023	1	864	-0.28	GI	NSB	3~6	E	L	Father	H	NR
Yang et al., 2024	6	1349	-0.04	GI	NSB	7~12	W	L	Father	H	48%
			-0.06	GI	NSB						
			-0.11	SI	NSB						
			-0.04	SI	NSB						
			0.16	NI	NSB						
			0.01	NI	NSB						
Yoon et al., 2024	22	2040	-0.07	GI	NSB	3~6	W	L	Parent	H	50.34%
			-0.05	GI	NSB						
			-0.12	GI	NSB						
			-0.04	GI	NSB						
			-0.02	GI	NSB						
			-0.02	GI	NSB						
			-0.05	GI	NSB						
			-0.03	GI	NSB						
			-0.08	GI	NSB						
			-0.06	GI	NSB						
			0.00	GI	NSB						
			0.00	GI	NSB						
			-0.04	SI	NSB						

			-0.03	SI	NSB						
			-0.08	SI	NSB						
			-0.07	SI	NSB						
			-0.06	SI	NSB						
			-0.02	SI	NSB						
			-0.01	SI	NSB						
			-0.04	SI	NSB						
			-0.04	SI	NSB						
			-0.03	SI	NSB						
Zhang et al., 2022	7	478	0.02	GI	PSB	3~6	E	H	Father	H	47.50%
			0.19	SI	PSB						
			0.15	SI	PSB						
			0.27	SI	PSB						
			0.28	SI	PSB						
			0.05	SI	PSB						
			0.03	NC	PSB						
Zhang et al., 2025	12	635	0.41	GI	PSB	3~6	E	M	Father	H	50.87%
			0.34	GI	PSB						
			-0.29	GI	NSB						
			-0.24	GI	NSB						
			-0.35	GI	NSB						
			-0.27	GI	NSB						
			0.42	SI	PSB						
			-0.30	SI	NSB						
			-0.35	SI	NSB						

			0.39	NC	PSB						
			-0.27	NC	NSB						
			-0.34	NC	NSB						

Database-Specific Search Strategies:

All searches were conducted in May 2025, with the following parameters:

1. Web of Science Core Collection (WoSCC)

- Retrieval period: 1970-01-01 to 2025-05-31
- Language: English
- Field tag: Topic (TS=)
- Search string:

TS=("paternal involvement" OR "father involvement" OR "father participation" OR "father engagement")
AND TS=("social behavior" OR "prosocial behavior" OR "aggressive behavior" OR "aggression" OR "withdrawal" OR "shyness" OR "shy")
AND TS=(children OR child OR children*)

2. Scopus

- Retrieval period: 1970-01-01 to 2025-05-31
- Language: English
- Field tag: Title/Abstract/Keywords (TITLE-ABS-KEY)
- Search string:

TITLE-ABS-KEY("paternal involvement" OR "father involvement" OR "father participation" OR "father engagement")
AND TITLE-ABS-KEY("social behavior" OR "prosocial behavior" OR "aggressive behavior" OR "aggression" OR "withdrawal" OR "shyness" OR "shy")
AND TITLE-ABS-KEY(children OR child)

3. PubMed

- Retrieval period: 1970-01-01 to 2025-05-31

- Language: English
- Field tag: Title/Abstract
- Search string:
("paternal involvement"[Title/Abstract] OR "father involvement"[Title/Abstract] OR "father participation"[Title/Abstract] OR "father engagement"[Title/Abstract])
AND ("social behavior"[Title/Abstract] OR "prosocial behavior"[Title/Abstract] OR "aggressive behavior"[Title/Abstract] OR "aggression"[Title/Abstract] OR "withdrawal"[Title/Abstract] OR "shyness"[Title/Abstract] OR "shy"[Title/Abstract])
AND ("children"[Title/Abstract] OR "child"[Title/Abstract])

4. PsycINFO (via EBSCOhost)

- Retrieval period: 1970-01-01 to 2025-05-31
- Language: English
- Field tag: All Text (TX)
- Search string:
TX ("paternal involvement" OR "father involvement" OR "father participation" OR "father engagement")
AND TX ("social behavior" OR "prosocial behavior" OR "aggressive behavior" OR "aggression" OR "withdrawal" OR "shyness" OR "shy")
AND TX (children OR child)

5. Wiley Online Library

- Retrieval period: 1970-01-01 to 2025-05-31
- Language: English
- Field tag: Title/Abstract/Keywords
- Search string:
("paternal involvement" OR "father involvement" OR "father participation" OR "father engagement")
AND ("social behavior" OR "prosocial behavior" OR "aggressive behavior" OR "aggression" OR "withdrawal" OR "shyness" OR "shy")
AND (children OR child)

6. China National Knowledge Infrastructure (CNKI)

- Retrieval period: 1970-01-01 to 2025-05-31

- Language: Chinese
- Field tag: 主题 (Topic)
- Search string:

主题=(父亲参与 OR 父亲投入 OR 父亲教养参与)
AND 主题=(社会行为 OR 亲社会行为 OR 攻击行为 OR 攻击性 OR 退缩 OR 羞怯 OR 害羞)
AND 主题=(儿童 OR 幼儿 OR 小学生)
